# Adrenocortical Carcinoma Presenting with Signs of Acute Abdomen

**DOI:** 10.1155/2013/132726

**Published:** 2013-02-03

**Authors:** Dimitrios Symeonidis, Ioannis Chatzinikolaou, Georgios Koukoulis, Ioannis Mamaloudis, Konstantinos Tepetes

**Affiliations:** Department of General Surgery, University Hospital of Larissa, Mezourlo, 41110 Larissa, Greece

## Abstract

*Background*. Adrenocortical carcinomas represent rare malignancies. In cases of hormone-secreting tumors, the hormone in excess determines the nearly diagnostic clinical presentation. Biologically inert tumors are diagnosed either due to the mass effect or incidentally. The purpose of the present study was to present an extremely rare presentation pattern of adrenocortical carcinoma. *Case Presentation*. We present the case of a 35-year-old female patient that underwent emergency laparotomy due to signs of acute abdomen and concomitant cardiovascular collapse caused by a spontaneously ruptured large adrenocortical carcinoma. *Conclusion*. Spontaneous rupture of an adrenocortical carcinoma presenting with signs of acute abdomen is an extremely rare clinical scenario. Increased level of suspicion is essential in order to diagnose and treat timely this life-threatening complication.

## 1. Introduction

Adrenocortical carcinomas represent rare malignancies with two to ten new cases diagnosed per one million inhabitants every year [1]. A dismal 5-year survival rate of 35% has been reported for these tumors [2]. The protean clinical manifestations of adrenal carcinomas render accurate diagnosis a challenging procedure. In cases of hormone-secreting tumors, the hormone in excess sets the scene and determines the nearly diagnostic clinical presentation. A relatively long-term history of symptoms suggestive of the culprit hormone overproduction is usually the rule in clinical practice [3]. On the other hand, biologically inert tumors are diagnosed either due to the mass effect or they are discovered incidentally during an abdominal imaging investigation performed for an irrelevant to the tumor indication [3]. 

Interestingly, even large noncortisol-producing adrenocortical carcinomas have only a minimal effect on patient's well-being complicating the diagnostic process [3]. On the other hand, the clinical pattern and symptom complex of acute abdomen is an extremely rare presentation of adrenocortical carcinoma. Spontaneous rupture or infarction of a large adrenal mass is the logical prerequisite for such phenomenon. A limited number of cases, less than ten to our knowledge, exhaust the available literature on the subject [4–10]. In the present study we present the rare case of a young female that underwent emergency laparotomy due to signs of acute abdomen and concomitant cardiovascular collapse caused by a spontaneously ruptured large adrenocortical carcinoma. 

## 2. Case Presentation

A 35-year-old, otherwise healthy, female patient was admitted in the emergency room department complaining of an excruciating abdominal pain reflecting at the back and lightheadedness over the last two hours. The onset of pain was suddenlly reaching a peak point over a couple of minutes and it was combined with diffuse perspiration and fatigue. Despite the fact that the patient reported a generalized sense of weakness, she remained alert and oriented. Upon arrival the patient had a BP of 80/55 mm Hg, 115 pulses/min, blood O_2_ saturation of 99%, and a body temperature of 37 degrees Celsius.

Physical examination revealed a relatively distended abdomen with absent bowel sounds. Marked tenderness was elicited during the deep palpation of the left lower abdominal quadrant and of the periumbilical area. The hallmarks of parietal peritoneal irritation, that is, tenderness on percussion and rebound tenderness, were present. Immediate intravenous assess was obtained and two large bore peripheral vein catheters were inserted in each forearm followed by the vigorous administration of crystalloid fluids. Blood samples were obtained for crossmatching and appropriate tests. Soon after, the administration of the first bolus of intravenous fluids the patient's hemodynamic status was stabilized with gradual normalization of vital signs. Urine human chorionic gonadotropin (h-CG) levels were within normal range and a gynecological consultation assisted by a transabdominal ultrasonographic assessment ruled out an occult pathology from the internal genitalia.

**Figure 1:**
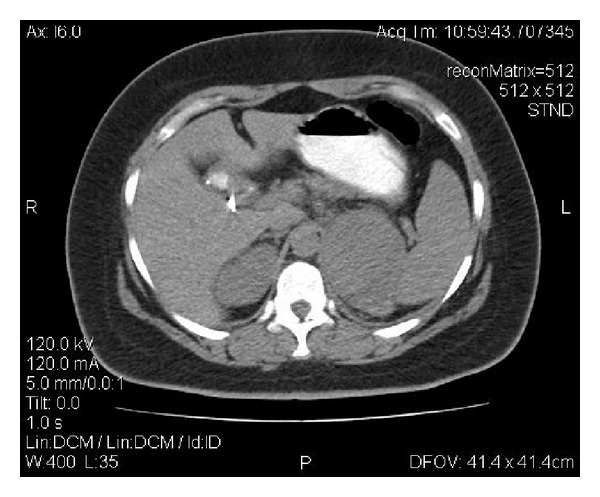
A CT scan image showing the adrenal tumor.

**Figure 2:**
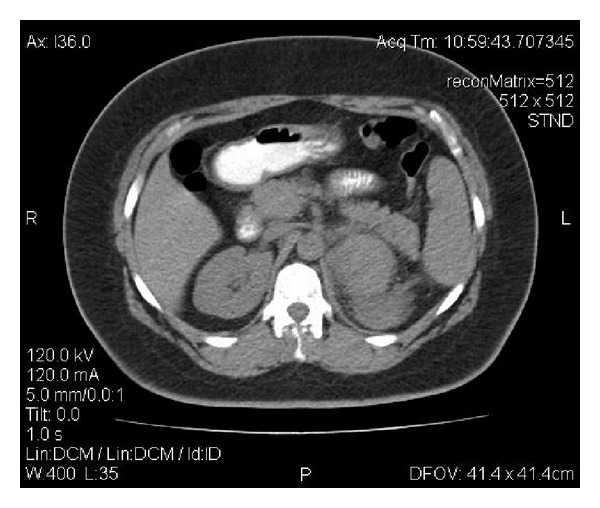
A CT scan image showing the adrenal tumor and the pertinent hematoma.

Under these circumstances, an emergency computed tomography (CT) scan of the abdomen with intravenous contrast media was then conducted. A 9 cm in diameter adrenal tumor surrounded by a large retroperitoneal hematoma was observed (see Figures 1 and 2). Active extravasation of the contrast media during the arterial phase of the CT scan denoted the continuous bleeding and underlined the need for emergency intervention. The tumor had the imaging characteristics of an adrenocortical carcinoma, that is, large size, heterogeneity, central necrosis, and irregular enhancement with increased concentrations of the contrast media in the periphery of the lesion. A minor intraperitoneal fluid collection especially in the Douglas's pouch was an additional imaging finding. 

**Figure 3:**
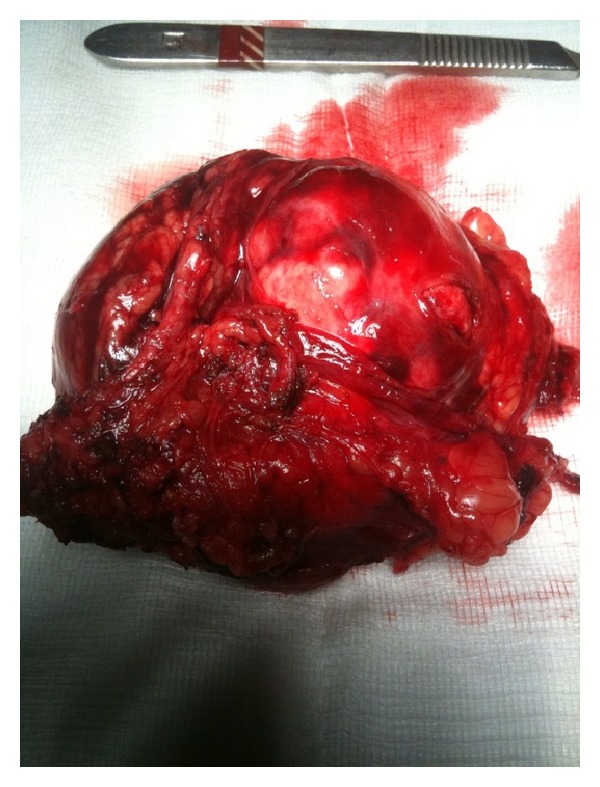
The surgical specimen.

An immediate operative approach was decided and the patient underwent a laparotomy with a midline vertical suprainfra umbilical incision. A thin serosanguineous fluid was evident throughout the peritoneal cavity while a bulging tensed mass consistent with a contained retroperitoneal hematoma was identified in the anatomic position of the left kidney. With careful maneuvers, the sheath of the hematoma was opened and after the evacuation of blood clots with copious irrigation the mass in question was identified just above the left kidney in close proximity to the splenic hilum and the pancreatic tail. The tumor was dissected free from the adjacent structures with mainly sharp dissection revealing a deep oblique rupture on tumor's surface and obvious actively bleeding vessels. An en-mass removal of the tumor (see Figure 3) was followed by careful hemostasis in the resulting cavity. The surgical specimen was then sent for complete pathology examination. No frozen section examination was conducted intraoperatively. Although there were no compelling pieces of evidence to suggest the use of drain, a silicon drain was finally left in place. The proximity of the pancreatic tail in an area of extensive dissections without at least initially adequate exposure due to the presence of the hematoma as well as the relatively objective need to monitor the drain output at least during the immediate postoperative period were the main arguments.

The patient had an uneventful postoperative recovery in the surgical department's clinic and was discharged from the hospital on the 7th postoperative day. The histopathological examination of the surgical specimen confirmed the imaging diagnosis of an adrenocortical carcinoma. Subsequently, the case was thoroughly discussed in the setting's multidisciplinary meeting where a complete staging with CT chest and abdomen was decided. The findings were unremarkable. No picese of evidence of metastatic disease were observed. Unfortunately, the follow-up period is rather limited to provide solid data regarding the long-term results. 

## 3. Discussion

Adrenocortical cancers are uncommon malignancies. Most patients, approximately 60%, present with signs emanating from hormone overproduction [3]. A rapidly progressing Cushing's syndrome sometimes combined with clinical signs of virilization is the most common presentation [1, 3]. The autonomously overproduced cortisol sets the stage for the classical manifestations and provokes the characteristic clinical syndromes. In female patients, androgen-secreting adrenal tumors result in virtualization with hirsutism, oligo-menorrhea, and alopecia [3]. Male patients with estrogen-secreting tumors develop gynecomastia and concomitant abnormalities of the external genitalia [3]. On the other hand, the hallmarks of primary hyperaldosteronism, that is, hypertension and hypokalemia, denote the presence of an aldosterone-secreting tumor [3].

Hormonally inactive adrenocortical carcinomas are usually associated with nonspecific symptoms such as back pain, abdominal discomfort, nausea, vomiting, and an ill-defined sense of abdominal fullness. The so-called mass effect represents the impact and the effects of tumor enlargement in adjacent structures [3]. Recently, the widespread use of abdominal imaging modalities created a new diagnostic category of mainly asymptomatic adrenal tumors referred to as incidentalomas. Tumor dimensions, certain imaging characteristics, and clinical history determine the malignant potential and dictate the proper management in these challenging cases [11]. Only occasionally patients with adrenocortical carcinoma present with fever, weight loss, and anorexia [12]. 

Endocrine assessment is of paramount importance in the initial elective workup of adrenal lesions. Certain hormone secretion patterns may predict the malignant potential of such tumors. Increased plasma levels of estradiol in male patients, high concentration of serum dehydro-epiandrostenedione, or detectable compounds of steroid precursors can stand as valuable cancer markers [3]. Imaging studies supplement the hormonal workup. Computed tomography (CT), magnetic resonance imaging (MRI), and recently positron emission tomography (PET) have been all utilized in an attempt to accurately distinguish benign from malignant adrenal tumors. Consistently, the size of an adrenal lesion represents the most important determinant of its biologic behavior [13]. Tumors more than six cm in diameter carry an increased risk of malignancy and thus require appropriate management [13].

In the present study we present an extremely rare clinical presentation of an adrenocortical carcinoma. The differential diagnosis of acute abdomen combined with hemodynamic instability in a young female patient in the absence of trauma classically includes pathologies emanating from the female reproductive system as well as intra-abdominal vascular catastrophes. Among the oncological/proliferating intra-abdominal entities that have been associated with the devastating presentation of spontaneous rupture, hepatic adenomas especially during pregnancy are the most frequent. However, the rare diagnosis of a spontaneously ruptured adrenal tumor that justified the patient's symptoms was surprisingly established after the emergent imaging investigation.

 Provided that an emergency presentation was the actual case, a prompt assessment and timely intervention were imperative. An emergency angiography with embolization would certainly be a valid option. Unfortunately, due to the logistics in our medical setting we do not access the angiography suite in the emergency setting. Within this framework, an open surgical approach was decided via a vertical midline laparotomy incision. Intraoperatively, when the source of hemorrhage was identified and controlled a proper oncological surgical resection of the ruptured adrenal tumor was successfully undertaken. Although drawing definite conclusions based on a single case is not only risky but also inappropriate, however, a logical assumption would be that the large size of the lesion could be considered as a predisposing factor for such kind of presentation [6]. 

In conclusion, spontaneous rupture of an adrenocortical carcinoma presenting with signs of acute abdomen is an extremely rare clinical scenario. Increased level of suspicion is essential in order to diagnose and treat timely this life-threatening complication.
